# A review of applications of automated ventricular parcellation from magnetic resonance imaging of the brain

**DOI:** 10.3389/fneur.2025.1639381

**Published:** 2026-04-01

**Authors:** Birra Taha, Alexandra Benson, Leopold Arko IV, Noam Harel, Carolina Sandoval Garcia, Daniel Guillaume, Robert McGovern

**Affiliations:** 1 Department of Neurosurgery, University of Minnesota, Minneapolis, MN, United States; 2 Normandale Community College, Minneapolis, MN, United States; 3 Department of Radiology, Center for Magnetic Resonance Research, University of Minnesota, Minneapolis, MN, United States

**Keywords:** ventricles, hydrocephalus, computer vision, segmentation, deep learning

## Abstract

Ventricular parcellation or segmentation is the systematic assignment of pixels (or voxels), from an image of the brain, to the ventricular compartment. As opposed to manual methods, automated techniques seek to streamline segmentation for better, objective delineation of the ventricles. The refinement of these methods, powered by advances in computer vision, has provided significant biological insight into the pathogenesis of many neurological diseases affecting both adults and children. In this article, we present a review of applications of automated ventricular segmentation from magnetic resonance imaging (MRI) and offer a brief primer on brain segmentation methods to non-technical readers.

## Introduction

Neuroimaging plays a crucial role in both the clinical and research spaces. The availability of imaging coupled with advances in both computer hardware (dedicated graphical processing units, growth in storage space, etc.) and computer vision algorithms have led to a paradigm shift in understanding neurological disease. The cerebral ventricles are an interconnected network of fluid-filled structures in the center of the brain and primarily serve as a reservoir for cerebrospinal fluid (CSF). The ventricular system plays a vital role in understanding both development and pathology in many neurological diseases. Imbalances between secretion and absorption classically lead to pathologic dilation of the ventricular system known as hydrocephalus. However, ventricular enlargement (VE) may also occur in many other situations. Brain segmentation refers to the precise labeling of pixels in an image as a structure of interest. Ventricular segmentation thus refers to classifying pixels as belonging to the ventricular space or not. Frequently, ventricular labeling/segmentation is seen as one of many tasks within whole-brain segmentation.

In neuroimaging research, brain segmentation has been a particularly popular subject of research for many years (1, 2). As a research and clinical tool, the ubiquity, rapidity, and relatively high resolution nature of magnetic resonance imaging (MRI) of the brain has made it the focal point of brain segmentation tasks (3–7). Manual segmentation is time consuming. The extensive time required from experts along with problems with inter- and intra-rater inconsistencies have made it less favorable as a technique. As a proxy for ventricular volume estimation from segmentations, approximations are often employed in the form of indices—some techniques dating as far back as the early 20th century using pneumoencephalography (8). Semi-automated and automated segmentation have been developed with advances in computer technology and have made the segmentation process less tedious. Automated ventricular segmentation is the precise labeling of pixels in the ventricles from background structures without requiring manual intervention (9). Semi-automated methods encompass a large swath of techniques where researchers will often streamline manual segmentation through machine assistance in some form (9). The introduction of MRI afforded clinicians a new perspective into accurate brain segmentation. The high signal-to-noise ratio as compared to computed tomography (CT) made it a perfect substrate for separating tissue composition using existing methods in computer vision (10). As derived by methods like these and more modern ones, accurate volumetric analysis from segmentations of key structures has had a pivotal role in deriving clinical insights into neuropathology. Automated segmentations of many cortical and subcortical structures have been used to interrogate their volumetric, texture, and shape differences in diseases like Alzheimer’s Disease (AD) (11), epilepsy (12), attention deficit/hyperactivity disorder (ADHD) (13), and numerous others (14, 15).

While there has been considerable interest in brain segmentation tasks in neuroimaging, little focus has been placed on the clinical applications in ventricular segmentation. This review briefly examines historical and modern methods in ventricular segmentation, discusses publicly available tools, and explores clinical applications. Given the vast literature across multiple modalities, the focus of the review is limited to magnetic resonance imaging (MRI) of the brain.

### A brief review of publicly available models

Despite the obvious clinical and research need for accurate, automated ventricular segmentation, most models described in the literature are not publicly available. In the computer vision literature, models with new architectures are often trained on publicly available data due to its accessibility (16–18). However, final pre-trained models are seldom shared. Developed from pivotal work by Dale and Fischl et al. in the 1990s, among other things, FreeSurfer provides high resolution whole-brain segmentation (19). FreeSurfer exists as an open source set of tools for analyzing neuroimaging data. Existing as a *de facto* industry standard, its research footprint on brain segmentation tasks is unparalleled. Numerous validation studies have proven its consistency and accuracy against manually derived structures (20). In its segmentation protocol, it provides labeling for the lateral ventricles, third ventricle, and fourth ventricle. Recently, a deep-learning-based analog for FreeSurfer titled FastSurfer has been proposed and has significantly reduced runtime for brain segmentation from 9.5 h to minutes, while providing mostly higher accuracy in most benchmarks (21).

Other publicly available non-machine learning based methods, such as MALPEM (22), volBrain (23), RUDOLPH (24), JLF (25), have shown promise in ventricular segmentation. However, when interrogating large datasets, they have fallen out of favor due to their lengthy runtimes. In contrast, QuickNAT and SLANT are two recently published and publicly available models relying on deep neural networks offering whole brain segmentation including sub-segmentation of the ventricles (26, 27). Both have fast runtimes (minutes) and have shown improved accuracy over other models. We include a non-exhaustive list of available models that include ventricular segmentation as a table in the Supplementary material.

### A primer on methods of segmentation

The task of a semantic segmentation algorithm in MRI is the assignment of voxel labels to the structure(s) of interest. Although the technical details of these algorithms are beyond the scope of this review, a broad overview of cutting-edge techniques in brain segmentation is provided. These include thresholding, clustering, statistical and probabilistic modeling (commonly Gaussian mixture models), edge detection (a more rudimentary approach), atlas-based methods, region-growing techniques, and deep learning-based approaches.

Interestingly, many of these methods have been used in various forms for decades. Early edge detection techniques were amongst the first considerations for automated ventricular segmentation from MRI–dating back to the late 80s and early 90s (28). More recently, deep neural networks have transformed the landscape of algorithmic imaging methods applied to semantic segmentation due to their fast, accurate, and uncanny ability to learn. While shortcomings with deep learning models are certainly present (29), their superiority over other methods in ventricular segmentation tasks is undeniable (30). Neural network models exist in various forms depending on their architectures (e.g., convolutional networks, recurrent networks, autoencoders, etc.). While traditionally considered “data-hungry,” newer architectures, particularly autoencoders, have challenged this notion in certain tasks (31, 32). In brain tumor segmentation challenges, U-Net architectures (a type of autoencoder network) have most recently shown high accuracy without significantly more memory requirements nor requiring significant numbers of training samples (32).

Across the literature of ventricular segmentation in MRI, there has been a clear focus on healthy adults, or adults with certain pathologies (33)—with a glaring paucity in models dedicated to children. In pediatrics, the development of these segmentation models relies on the consideration of their unique complexities related to neurodevelopment. Accounting for age-related changes secondary to myelination and the development of cortical and subcortical structures is extremely difficult (34). These considerations have proven challenging for many existing models which have limited their transferability to children (35). Moreover, these limitations are further reinforced in the setting of pathology where severe anatomical alterations and deformations exist.

#### Thresholding based methods

**Figure 1:**
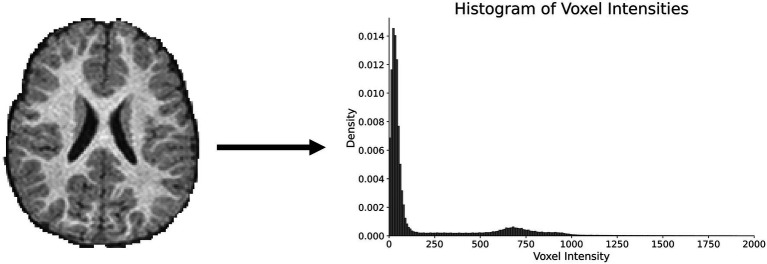
A sample MRI of the brain with intensity values represented as a frequency histogram.

**Figure 2:**
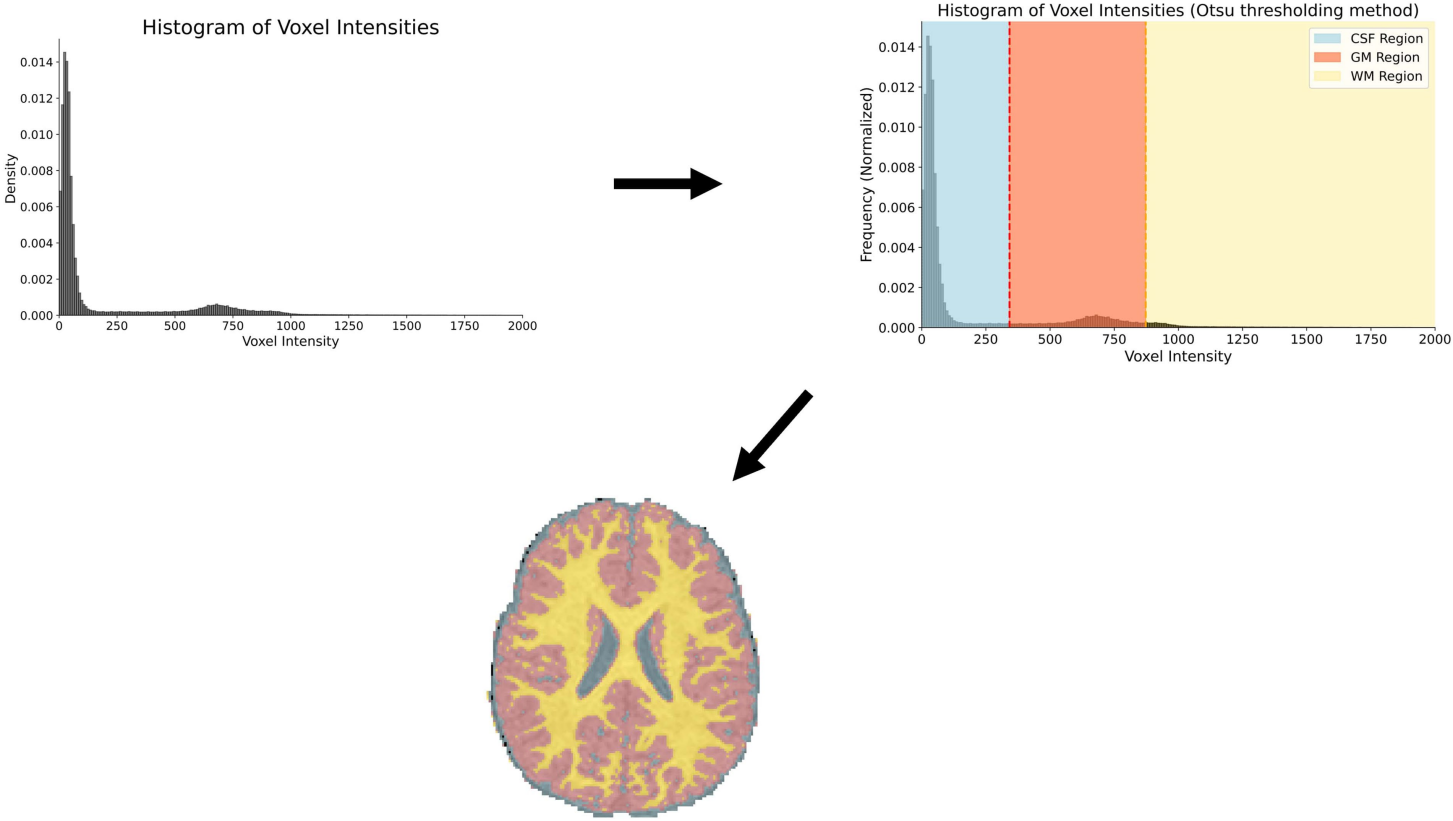
An example using otsu thresholding of intensity values to define cut-off values. Once tissue classes are determined, each voxel is colored by its membership to a specific class. In this example, three tissue types are identified: CSF (blue), white matter (yellow), gray matter (red).

Thresholding methods begin by analyzing voxel intensities across different tissue types. Based on these intensity variations, cutoff values are established to classify voxels and assign corresponding tissue labels. Optimizing threshold values for tissue types is an ongoing problem in brain segmentation (36–39). This method is less effective for ventricular segmentation due to its inability to distinguish intraventricular CSF from CSF in the subarachnoid space. As a result, it is primarily used as an adjunct in semi-automated pipelines for ventricular segmentation. Figure 1 demonstrates a typical histogram plot for intensity values from a healthy brain MRI. Figure 2 illustrates a commonly used method for thresholding intensity values (40).

#### Statistical methods (Gaussian mixture models)

**Figure 3:**
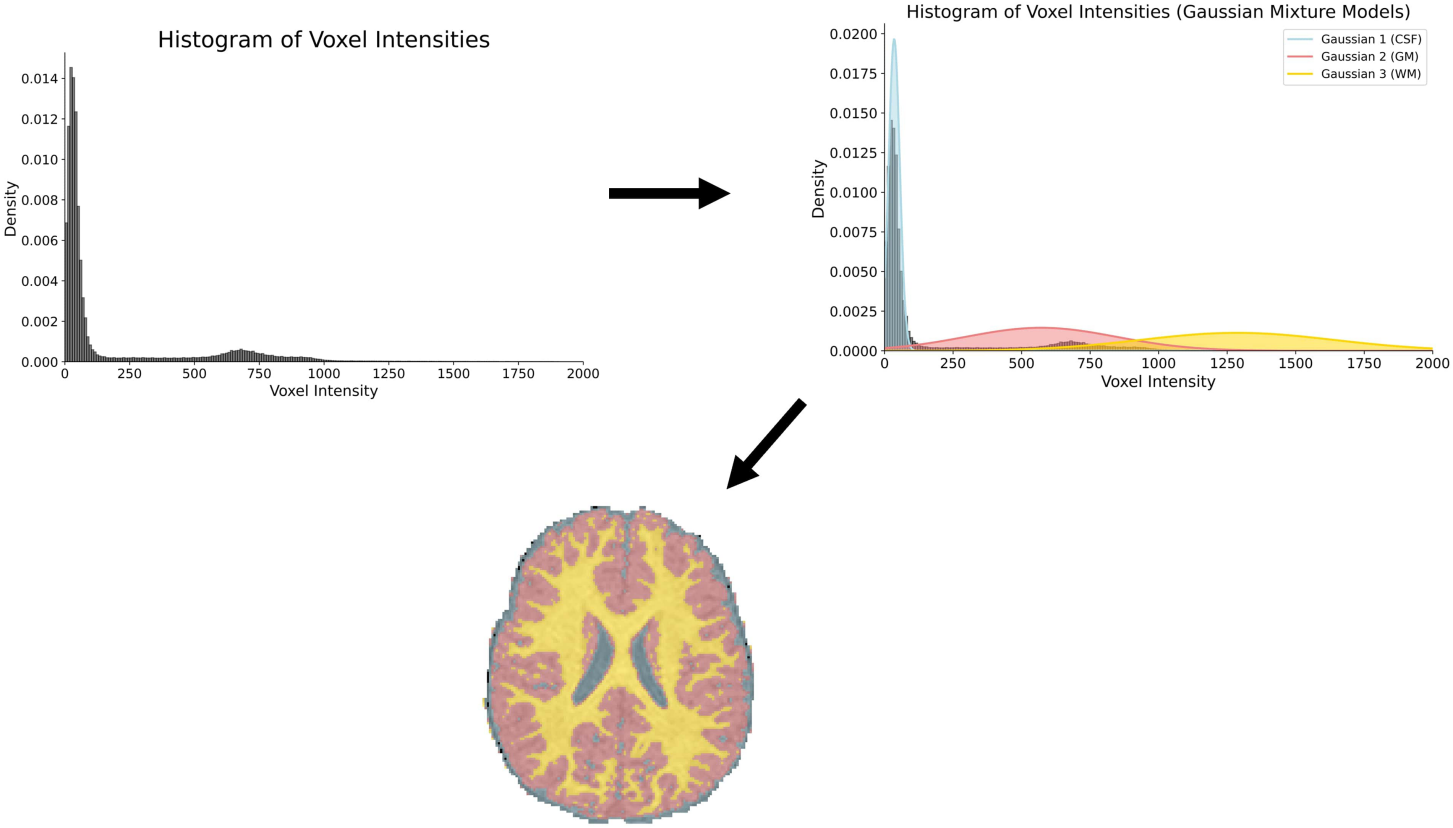
Using voxel intensity values, a gaussian mixture model (GMM) is fit to the distribution. Voxels are subsequently labeled based on their highest ranked membership to one of the classes.

Using voxel intensities, a probability distribution is fit to the model. A segmentation model using Gaussian mixture model (GMM) assumes the intensity distribution is derived from multiple Gaussian distributions (mostly from their biological origins). Using this kind of probabilistic labeling, pixels are classified based on their probability of membership to one class (one of the Gaussians) obtained via the expectation–maximization algorithm (41). Amongst the probabilistic models, GMMs are among the most popular by themselves (42, 43), as part of a combined (44) or extended approach (45). By their construction, GMMs alone cannot distinguish ventricular CSF and CSF in the subarachnoid space. For this reason, incorporation of prior spatial organization into GMMs can be used to boost performances. Overall, however, probabilistic approaches have less than optimal performance and struggle to reach Dice Similarity Coefficients (DSC) greater than 0.7. We show a pictorial representation of a GMM in Figure 3.

#### Region/seed-growing

**Figure 4:**
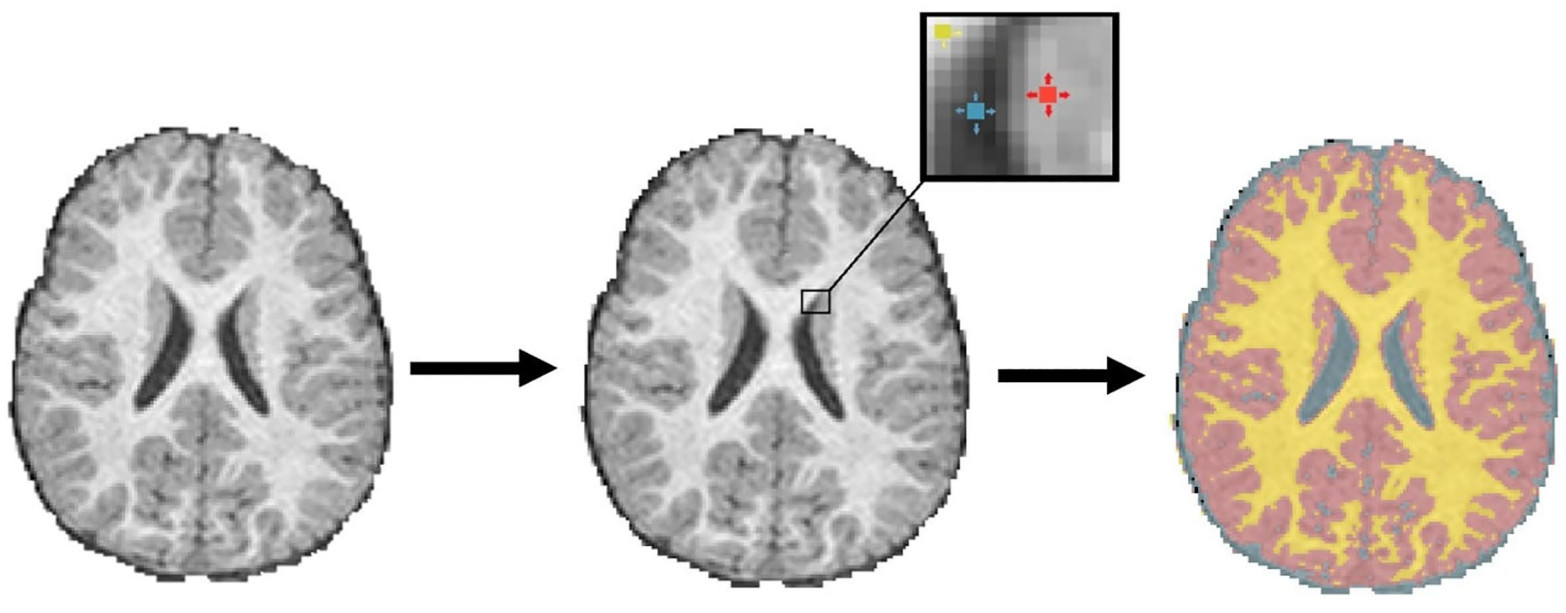
Using seed points, a region “grows” iteratively by labeling nearby pixels with similar intensities to define tissue/class membership.

Region/seed-growing methods use voxel intensity information and aid from an operator. A single voxel or small cluster of voxels act as a “seed point” and iteratively label neighboring voxels based on similarities in their intensity. In this way, the regions “grow” and contour to the structure of interest. In ventricular segmentation, given the stark intensity differences between CSF and neighboring white matter (WM) and grey matter (GM), region-growing methods have historically shown acceptable performances (46–48). However, these methods struggle when the region is farther apart or disconnected. Figure 4 highlights its capabilities on a sample image.

#### Traditional machine learning approaches

##### Clustering

Clustering is part of the family of unsupervised learning methods. These techniques include clustering by k-means, fuzzy c-means, and numerous others. Using voxel intensity values, the k-means clustering algorithm uses optimization techniques to create data centroids (49). In doing so, samples are labeled according to their proximity to the nearest centroid. Fuzzy c-means clustering generalizes K-means by allowing more than one membership to a centroid (with a ‘membership value’) and iteratively updating the centroids and membership weights until reaching stability. Clustering methods can perform adequately in CSF segmentation tasks (50, 51), or can be combined with other techniques (52). However, in modern use, clustering methods for CSF/ventricular segmentation solve the tissue classification problem as part of one step in a larger whole-brain segmentation pipeline (53). Random forests are an ensemble method that seek consensus agreement from many decision trees. Random forests have been used in ventricular segmentation (54), but have had more popularity in brain tumor segmentation (55, 56).

##### Surface-based techniques

Deformable models are the workhorse of surface-based methods. In their design, they work by iterative evolution of initial contours around an object of interest until the contour aligns with the object’s boundaries (57). The rate and accuracy of the evolution of the contour depends most importantly on sharp edges (i.e., large differentials in voxel intensity) defining your boundary and minimal noise. Contour evolution seeks to minimize an energy function which takes voxel intensity, the ‘smoothness’ of the contour, and the intensity differential between neighboring voxels (58).

##### Atlas-based methods

Atlases are pre-labeled brain templates obtained from brain MRIs averaged from normal brains. Early, pioneering work in stereotactic targeting of deep brain nuclei by Talairach et al. (59) relied on a single subject. The current, most popular template, named ‘ICBM152’ was developed by the Montreal Neurological Institute (MNI) from 152 brains in healthy controls (60). In atlas-based methods, using mathematical transforms, a subject’s image is aligned to the atlas in a process known as “registration.” The exact transforms used have been extensively studied with each having their strengths and weaknesses (61–64). In their work, Cabezas et al. provide an in-depth review of atlas-based methods (65). The use of atlases has been a mainstay in ventricular segmentation (65, 66) but can be challenging in the setting of minor or major pathology (67) variations (68).

##### Deep-learning based methods

**Figure 5:**
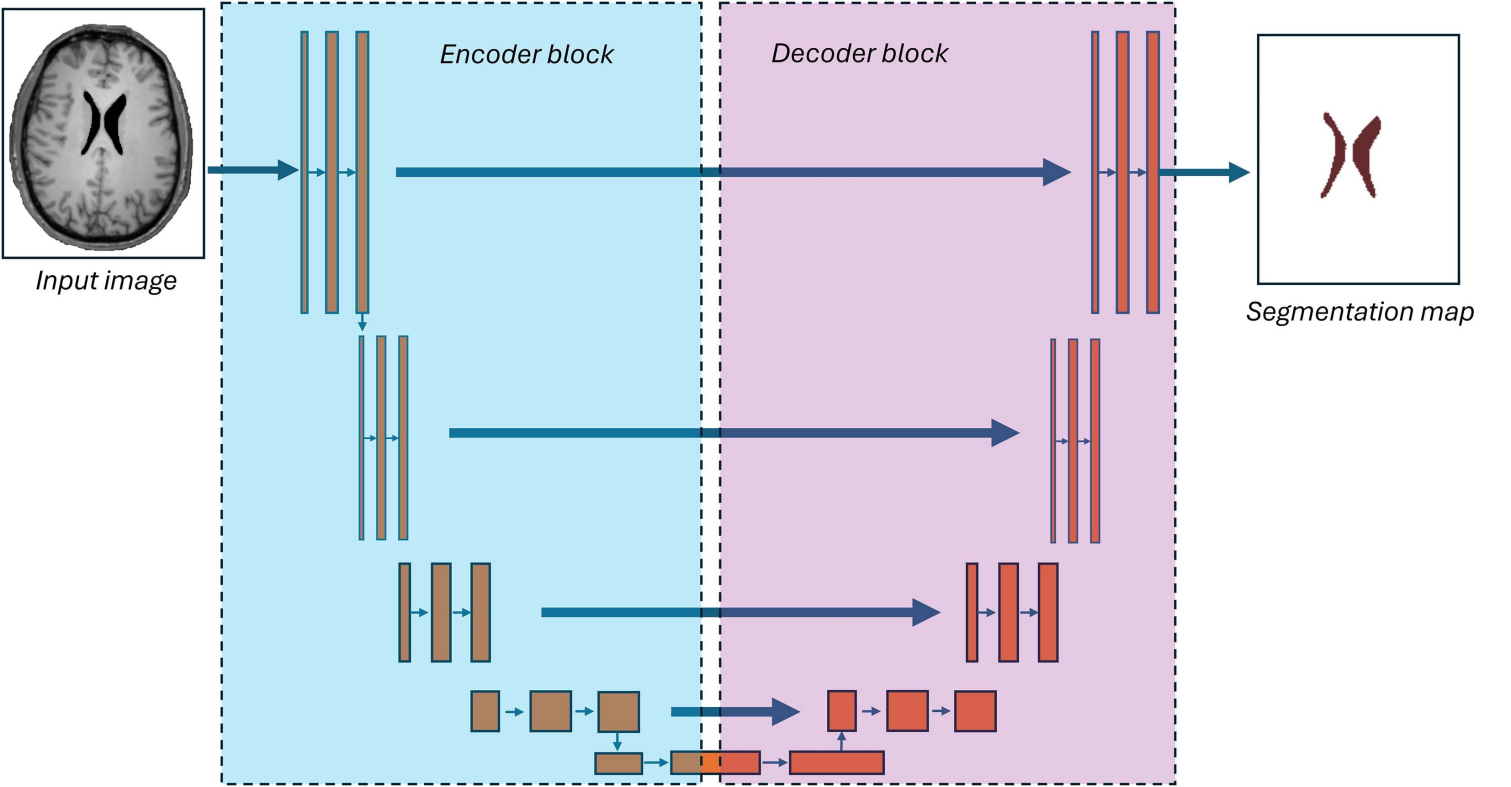
From an input MR image, a U-net model sends the image into a low-dimensional embedding (“the encoder”) then subsequently recovers the image segmentation map (“the decoder”).

Neural networks are constructs which consist of nodes organized in layers and are connected by edges. Nodes are analogous to neurons in the brain and propagate their input forward to its connected neighboring nodes. In practice, nodes receive a summed, weighted input which then goes through an activating function before being passed onto the next layer. See previous work by Fawzi et al. (69) for more details. A U-Net architecture has been one of the most successful neural network frameworks for brain segmentation and consists of an encoder unit, decoder unit, and final output layer. U-Net models have performed particularly well in whole-brain segmentation tasks (including the ventricles) (21, 26). They also demonstrate superior performance in the setting of brain tumor segmentations (70, 71). A sample architecture is illustrated in Figure 5.

### Ventricular segmentation in adults

#### Aging and NPH

Early cross-sectional studies in healthy patients have shown age-related and gender differences in brain volumes. Large-scale automated whole-brain segmentations performed on non-pathological populaces have revealed the lateral ventricles as having the most pronounced aging effects (72). In one study, when combined with periventricular white matter segmentations, subcompartment analysis derived from segmentations of the lateral ventricle showed the occipital horns as the most pronounced location for edema in cognitively impaired individuals (73).

Normal pressure hydrocephalus (NPH) is a syndrome defined as a triad of urinary incontinence, dementia (typically memory loss), and a magnetic, shuffling gait (74). Radiographically, NPH is seen with ventriculomegaly (75). While lumbar puncture and lumbar drain trials play an important role in determining who may be suitable candidates for shunt placement, each have their diagnostic limitations (76). For this reason, shunt-responsiveness is often considered the gold-standard method for NPH diagnosis (77). This has driven the exploration of imaging techniques and segmentation to better identify patients who would benefit from treatment.

Researchers have utilized automated ventricular segmentation, sometimes by compartment, for large-scale volumetric studies comparing normal pressure hydrocephalus (NPH) to control patients and those with Alzheimer’s disease (78). Linear indices such as Evans Index have had reasonable success in identifying NPH patients (79). However, more recently, the anteroposterior diameter of the lateral ventricle index has shown potential as a more reliable metric (80). In their article, Kang et al. calculated voxel-wise correlations from lateral ventricle expansion with associated overlying cortical thinning in patients with NPH. They hypothesized compartment-specific pressure gradients within the lateral ventricle may play a role, and found the inferior portion of the bilateral lateral ventricles appeared much less affected in NPH patients (81). In addition, post-shunting third ventricular volume decrease was shown to be inversely correlated with scores on neuropsychiatric testing (82). Persistently decreased ventricular volume months after shunt treatment was also revealed via automated means by Cogswell et al. (83).

#### Psychiatric diseases

Neuroimaging studies of patients with schizophrenia (SZ) have identified numerous cortical and subcortical changes as compared to healthy controls. Ventricular enlargement (VE) in SZ was known in the mid 20th century--uncovered by pneumoencephalography (84). These findings were recapitulated with the advent of computed tomography (CT) in the 1970s (85) and eventually MRI in the 1980s (86). Current understanding of chronic schizophrenia has shown VE to be the most consistent radiographic finding (87). However, morphological changes in the brain have been well characterized in early phase SZ (88). Deciphering the pathogenesis of VE in SZ centers on an ex-vacuo hypothesis–whether at the cortical, subcortical, or subventricular zone level. Chung et al. showed cortical gray matter thinning appears to follow ventricular enlargement, with both widespread and focal areas of cortical loss in prodromal youth who develop psychosis (89). Dedicated morphometric studies using ventricular shape have uncovered differences in lateral ventricle configuration in the posterior region between affected and unaffected groups (90). Ventricular segmentations themselves can also be fed into prediction models. In their paper, Manohar et al. trained a neural network using raw ventricular segmentations, encoding both shape characteristics and volume information (including in all compartments), to successfully detect schizophrenia (91). Significant changes in lateral ventricle shape have been characterized following electroconvulsive therapy in patients with major depression disorder (92). Ventricular segmentation has also revealed chronic medication use can induce changes in ventricular morphometry. Selective serotonin reuptake inhibitor (SSRI) treatment has also shown to decrease both left and right lateral ventricular volumes (93).

Structural asymmetries also appear to play a role in many psychiatric diseases. A larger lateral ventricle asymmetry was negatively correlated with age of onset in schizophrenia. In their paper, Buschbaum et al. denote a left-minus-right ventricle size difference that was statistically greater in schizophrenia patients compared to healthy controls and schizotypal patients. Despite having been published 30 years ago, they use a semi-automated approach in segmentation with high intraclass correlation coefficient (0.979) (94). Automated methods have taken compartmental views of the lateral ventricle to uncover possible localized effects on surrounding brain regions. Moreover, focal effects of enlargement on key regions may drive hemispheric asymmetries related to mood regulation and emotion processing. The laterality index is defined as (L-R)/(L+R), where L is the left lateral ventricle volume and R is the right lateral ventricle volume, attempts to capture left–right asymmetry–where small values point to more symmetry and large values point to asymmetry (95). An inverse correlation has been noted between laterality index and areas involved with memory (96). Imbalance in cortical myelin content in the cingulate, frontal, and sensorimotor cortices (estimated from T1w/T2w ratios) were associated with ventriculomegaly and asymmetry (96). These findings point to a likely local dysregulation in conduction in periventricular cortical regions–in line with previously reported altered functional connectivity (97). In another article, using automated methods, asymmetric ventricular enlargement was theorized to play a role in occipital bending in patients with major depressive disorder (98). This lateral ventricular asymmetry was not seen in patients with bipolar disorder–noted to be possibly related to symmetric right lateral ventricle dilation from fluctuations between mania and depression (96).

#### Dementia—Alzheimer’s’ disease, frontotemporal dementia, mild cognitive impairment

Ex-vacuo ventricular dilatation is a consequence of normal brain atrophy. However, precise definitions of pathological enlargement in the setting of cognitive disorders such as Alzheimer’s Disease (AD), is an open problem. Significant evidence exists supporting ventricular volume as a proxy for capturing AD progression/severity (99, 100). In their 2008 paper, using a seed-based region-growing algorithm, Nestor et al. showed patients with AD had a nearly four-fold increase in ventricular volume as compared to normal elderly patients over a 6-month interval (101). They also show a unique ventricular volume trajectory in patients carrying an ApoE4 allele-seen and replicated in subsequent works (100). Lateral ventricle volumes appear to significantly correlate and predict other clinical metrics in dementia. Automated extraction of lateral ventricle volume was used to show ventricular volume can predict response inhibition (a core component of executive function) (102). In another study, a deep learning based approach was used to obtain planimetric measurements from segmentations of the third ventricle and frontal horns to help delineate patients with progressive supranuclear palsy (PSP) (103). In medial temporal lobe atrophy associated with Alzheimer’s disease, researchers validated an automated method for measuring hippocampal-to-ventricle ratio (HVR), as captured by hippocampal segmentation and lateral ventricle sub-segmentation of the temporal horns (104).

#### Neuro-oncology

In neurogenesis, the subventricular zone (SVZ) is considered a stem cell niche from which radial migration occurs. In glioblastoma, it has been shown that tumors in proximity to the SVZ show increased stem-like properties, a more aggressive clinical course, and worse survival (105). Steed et al. used the tumor segmentation to calculate precise distances from the tumor centroid to the ventricular border. In another novel application, researchers compared the centroid calculated from the ventricular segmentation to the centroid from its mapped template to capture a three-dimensional displacement vector describing mass effect in glioblastoma (106). Automated methods also hold promise in quantifying subtle volumetric changes in communicating hydrocephalus from patients with brain tumors (107).

### Pediatrics

#### Neurodevelopment

In brain segmentation tasks, fetal and neonatal imaging presents a unique challenge as compared to adults. Myelin content increases and shows changes in compactness in the developing neonatal brain which manifest as shortening of T1 and T2 relaxation times on MRI (108). As myelin content increases, changes in tissue intensity decrease signal-to-noise ratio–challenging the flexibility of most segmentation models using MRI. As such, dedicated segmentation models for fetal and neonatal ventricular segmentation have been created. Automated brain parcellation methods (including ventricles) specifically trained on fetal and neonatal brains have the ability to map developmental trajectories across the entire lifespan, identify abnormalities with higher power, and spur new hypotheses (34, 109, 110). Recently, using a mixture of automated and semi-automated methods, reference curves/centile curves for ventricular growth in children have been published (111). Xenos et al. (112) showed ventricular growth appears sexually dimorphic until the age of 6, with subsequent stable ventricular/intracranial volume ratios. Nonetheless, due to the low cost, strong clinical indications, and ease-of-use, most of the literature regarding development in neonates and infants is in large cranial ultrasonography studies (113, 114), including in preterm cases (115). This pervasiveness of use has led to dense literature creating nomograms and centile curves for these populations.

#### Hydrocephalus

Intraventricular hemorrhage is a relatively common finding in very low birth weight preterm neonates (116). Roughly 30%–50% of infants with severe intraventricular hemorrhage develop posthemorrhagic ventricular dilatation (PHVD)—with 20%–40% of those patients subsequently developing hydrocephalus (116, 117). Gholipour et al. (118) applied automated ventricular segmentation to fetal MRI scans of brains with ventricular enlargement, although they did not specify the presence or absence of hemorrhage. Subsequent work further extended to infants with accurate automated ventricular parcellations in infants with PHH (119). Using a 2D U-net, Quon et al. trained a deep neural network to segment the ventricles in pediatric patients with hydrocephalus from neonates to age 19 (120). Furthermore, significant literature exists in predicting the need for chronic CSF diversion in both ultrasound (121) and CT (122). Third ventricle morphometry is also an area of active research in hydrocephalus. Linear measurements derived from third ventricular segmentations were used to show the posterior portion to be particularly affected as well as to define volume thresholds for the determination of pathological dilatation (greater than 3 cm) (123).

#### Autism

Autism spectrum disorder (ASD) is characterized by persistent difficulties in social communication and interactions, as well as repetitive patterns of behavior, interests, or activities (124). Early diagnosis and subsequent behavioral interventions are pivotal. Using FreeSurfer applied to 81 participants (and their age and gender matched controls), Shiohama et al. (125) identified smaller bilateral nucleus accumbens and enlarged ventricles as possible biomarkers for the prediction of early ASD.

#### Future applications/discussion

While significant work has been done at the 1.5 T and 3 T level, newer deep learning models seek to utilize the higher resolution and better signal-to-noise ratio (SNR) afforded by 7-Tesla MRI (126–128). CEREBRUM-7 T is an end-to-end brain segmentation that relies on an underlying deep convolutional network to segment the brain and ventricles from 7 T MRI (129). As 7 T becomes more routine in surgical/stereotactic planning, future work will likely incorporate exact three-dimensional ventricular coordinates obtained from segmentations when calculating trajectories to the deeper, subcortical nuclei.

Given the increased abundance of imaging data, there has been considerable revived interest in the use of imaging-based morphometrics in understanding neurosurgical pathology. Confidently diagnosing these pathologies with imaging alone would obviate the need for invasive measures. For example, despite their minimally invasive nature, stereotactic biopsy of brain tumors carries a perioperative hemorrhage risk as high as 59% in malignant gliomas (130). Moreover, stereotactic sampling carries a known non-diagnostic sample rate of more than 20% (131). Newer indices have been derived and tested in predicting outcomes, identifying pathology, and even monitoring progression. For instance, anteroposterior lateral ventricular index, a new linear heuristic which incorporates information from the anterior–posterior length, has been used to capture patients with NPH (80). Similarly, fourth ventricular enlargement/bowing, captured by the angle of the roof, has been proposed as a predictor of surgical outcomes for Chiari malformation (132)—although with conflicting results (133). These works have spurred new considerations into understanding the association between ventricular morphometry and surgical outcomes (134). Conveniently, these measures can be rapidly derived from their segmentations (135). Callosal angle, a highly sensitive and specific marker for NPH (136), may be similarly derived using the lateral ventricle segmentation. In an analogous way, for any potential geometric descriptor with suspected association with a clinical variable, reference curves may be automatically generated and pathological patients compared to their age and gender-matched controls.

In pediatric patients with hydrocephalus, predicting who will benefit from receiving endoscopic third ventriculostomy as opposed to ventriculo-peritoneal shunting has also proven to be challenging. In their landmark paper by Kulkarni et al. (137) a predictive “success score” was created. However, finding a durable, radiographic supplement to this clinical scoring system has not yet been successful (138–140).

Despite the tremendous interest, most radiographic descriptors in outcome prediction rely on qualitative, binary labels (i.e., presence of bowing of the floor of third ventricle, displacement of lamina terminalis, etc). In doing so, researchers unintentionally discard vital, quantitative information such as third ventricular shape, curvature and spatial geometry. Rather than validating physician-derived indices, an objective approach using computational methods to “mine” indices could offer valuable insights. Framing this as an optimization problem, such as identifying indices with the strongest correlation to clinical outcomes, may improve predictive accuracy. For example, from pivotal, early work by O’Hayon et al. (141), frontal-occipital horn ratio (FOHR) is known to have strong correlation with ventricular volume in healthy (135) and pathological cases (142). An unbiased data mining approach may reveal previously undiscovered indices with stronger correlates for ventricular volume. Similarly, finding optimal third ventricular morphometrics to risk stratify endoscopic third ventriculostomy candidates is an area of active research (140), and would be ripe for index mining algorithms. In doing so, a computer algorithm would place the object in a three-dimensional coordinate system, and calculate pairwise distances between any two points on the object’s surface (if calculating a linear index). An objective, data-driven approach in this manner could spur the creation of newer morphometric indices and has clear applications as a metaheuristic. This approach of “hunting” for optimized strategies is not new; metaheuristic techniques have long been applied in computer vision tasks, including neuroimaging (143–145).

Interest in automated methods in ventricular segmentation continues to accelerate with significant pace with an enlarging footprint in the scientific literature. The continuous improvement of computer hardware will continue to drive the creation of better tools and techniques for ventricular segmentation.

## References

[1] Smith ML Smith LN Hansen MF. The quiet revolution in machine vision - a state-of-the-art survey paper, including historical review, perspectives, and future directions. Comput Ind. (2021) 130:103472. doi: 10.1016/j.compind.2021.103472

[2] Akkus Z Galimzianova A Hoogi A Rubin DL Erickson BJ. Deep learning for brain MRI segmentation: state of the art and future directions. J Digit Imaging. (2017) 30:449–59. doi: 10.1007/s10278-017-9983-4, 28577131 PMC5537095

[3] Knickmeyer RC Gouttard S Kang C Evans D Wilber K Smith JK . A structural MRI study of human brain development from birth to 2 years. J Neurosci. (2008) 28:12176–82. doi: 10.1523/JNEUROSCI.3479-08.2008, 19020011 PMC2884385

[4] Hua L Gu Y Gu X Xue J Ni T. A novel brain MRI image segmentation method using an improved multi-view fuzzy -means clustering algorithm. Front Neurosci. (2021) 15:662674. doi: 10.3389/fnins.2021.662674, 33841095 PMC8029590

[5] Lee B Yamanakkanavar N Choi JY. Automatic segmentation of brain MRI using a novel patch-wise U-net deep architecture. PLoS One. (2020) 15:e0236493. doi: 10.1371/journal.pone.0236493, 32745102 PMC7398543

[6] Igual L Soliva JC Hernández-Vela A Escalera S Jiménez X Vilarroya O . A fully-automatic caudate nucleus segmentation of brain MRI: application in volumetric analysis of pediatric attention-deficit/hyperactivity disorder. Biomed Eng Online. (2011) 10:105. doi: 10.1186/1475-925X-10-105, 22141926 PMC3252254

[7] Chupin M Hammers A Liu RSN Colliot O Burdett J Bardinet E . Automatic segmentation of the hippocampus and the amygdala driven by hybrid constraints: method and validation. Neuroimage. (2009) 46:749–61. doi: 10.1016/j.neuroimage.2009.02.013, 19236922 PMC2677639

[8] Evans WA. An encephalographic ratio for estimating the size of the cerebral ventricles. Am J Dis Child. (1942) 64:820. doi: 10.1001/archpedi.1942.02010110052006

[9] Trimpl MJ Primakov S Lambin P Stride EPJ Vallis KA Gooding MJ. Beyond automatic medical image segmentation-the spectrum between fully manual and fully automatic delineation. Phys Med Biol. (2022) 67:12TR01. doi: 10.1088/1361-6560/ac6d9c, 35523158

[10] Vannier MW Butterfield RL Jordan D Murphy WA Levitt RG Gado M. Multispectral analysis of magnetic resonance images. Radiology. (1985) 154:221–4. doi: 10.1148/radiology.154.1.3964938 3964938

[11] Chupin M Gérardin E Cuingnet R Boutet C Lemieux L Lehéricy S . Alzheimer’s Disease Neuroimaging Initiative, fully automatic hippocampus segmentation and classification in Alzheimer’s disease and mild cognitive impairment applied on data from ADNI. Hippocampus. (2009) 19:579–87. doi: 10.1002/hipo.20626, 19437497 PMC2837195

[12] Winston GP Cardoso MJ Williams EJ Burdett JL Bartlett PA Espak M . Automated hippocampal segmentation in patients with epilepsy: available free online. Epilepsia. (2013) 54:2166–73. doi: 10.1111/epi.12408, 24151901 PMC3995014

[13] Igual L Soliva JC Escalera S Gimeno R Vilarroya O Radeva P. Automatic brain caudate nuclei segmentation and classification in diagnostic of attention-deficit/hyperactivity disorder. Comput Med Imaging Graph. (2012) 36:591–600. doi: 10.1016/j.compmedimag.2012.08.002, 22959658

[14] Germann J Gouveia FV Martinez RCR Zanetti MV de Souza Duran FL Chaim-Avancini TM . Fully automated habenula segmentation provides robust and reliable volume estimation across large magnetic resonance imaging datasets, suggesting intriguing developmental trajectories in psychiatric disease. Biol Psychiatry Cogn Neurosci Neuroimaging. (2020) 5:4. doi: 10.1016/j.bpsc.2020.01.004, 32222276

[15] Najdenovska E Tuleasca C Jorge J Maeder P Marques JP Roine T . Comparison of MRI-based automated segmentation methods and functional neurosurgery targeting with direct visualization of the Ventro-intermediate thalamic nucleus at 7T. Sci Rep. (2019) 9:1119. doi: 10.1038/s41598-018-37825-8, 30718634 PMC6361927

[16] LaMontagne PJ Benzinger TLS Morris JC Keefe S Hornbeck R Xiong C . OASIS-3: longitudinal neuroimaging, clinical, and cognitive dataset for normal aging and Alzheimer disease. bioRxiv. (2019). doi: 10.1101/2019.12.13.19014902

[17] Marcus DS Wang TH Parker J Csernansky JG Morris JC Buckner RL. Open access series of imaging studies (OASIS): cross-sectional MRI data in young, middle aged, nondemented, and demented older adults. J Cogn Neurosci. (2007) 19:1498–507. doi: 10.1162/jocn.2007.19.9.1498, 17714011

[18] Marcus DS Fotenos AF Csernansky JG Morris JC Buckner RL. Open access series of imaging studies: longitudinal MRI data in nondemented and demented older adults. J Cogn Neurosci. (2010) 22:2677–84. doi: 10.1162/jocn.2009.21407, 19929323 PMC2895005

[19] Fischl B. FreeSurfer. Neuroimage. (2012) 62:774–81. doi: 10.1016/j.neuroimage.2012.01.021, 22248573 PMC3685476

[20] Tae WS Kim SS Lee KU Nam E-C Kim KW. Validation of hippocampal volumes measured using a manual method and two automated methods (FreeSurfer and IBASPM) in chronic major depressive disorder. Neuroradiology. (2008) 50:569–81. doi: 10.1007/s00234-008-0383-9, 18414838

[21] Henschel L Conjeti S Estrada S Diers K Fischl B Reuter M. FastSurfer - a fast and accurate deep learning based neuroimaging pipeline. Neuroimage. (2020) 219:117012. doi: 10.1016/j.neuroimage.2020.117012, 32526386 PMC7898243

[22] Ledig C Heckemann RA Hammers A Lopez JC Newcombe VFJ Makropoulos A . Robust whole-brain segmentation: application to traumatic brain injury. Med Image Anal. (2015) 21:40–58. doi: 10.1016/j.media.2014.12.003, 25596765

[23] Manjón JV Coupé P. Volbrain: an online MRI brain volumetry system. Front Neuroinform. (2016) 10:30. doi: 10.3389/fninf.2016.00030, 27512372 PMC4961698

[24] Carass A Shao M Li X Dewey BE Blitz AM Roy S . Whole brain Parcellation with pathology: validation on Ventriculomegaly patients. Patch Based Tech Med Imaging. (2017) 10530:20–8. doi: 10.1007/978-3-319-67434-6_3 PMC5815833 29459902

[25] Wang H Yushkevich PA. Multi-atlas segmentation with joint label fusion and corrective learning-an open source implementation. Front Neuroinform. (2013) 7:27. doi: 10.3389/fninf.2013.00027, 24319427 PMC3837555

[26] Guha Roy A Conjeti S Navab N Wachinger C. Alzheimer’s Disease Neuroimaging Initiative, QuickNAT: a fully convolutional network for quick and accurate segmentation of neuroanatomy. Neuroimage. (2019) 186:713–27. doi: 10.1016/j.neuroimage.2018.11.042, 30502445

[27] Huo Y Xu Z Xiong Y Aboud K Parvathaneni P Bao S . 3D whole brain segmentation using spatially localized atlas network tiles. Neuroimage. (2019) 194:105–19. doi: 10.1016/j.neuroimage.2019.03.041, 30910724 PMC6536356

[28] Langkowski JH Palmié SG von Koschitzky H Imme M Maas R Schmidt KH . Quantitative volumetric determinations on MR tomograms in communicating hydrocephalus. Rofo. (1989) 150:125–9. doi: 10.1055/s-2008-1046990, 2537503

[29] Nguyen A. Yosinski J. Clune J., Deep neural networks are easily fooled: high confidence predictions for unrecognizable images, arXiv [Preprint] (2014). Doi: 10.48550/ARXIV.1412.1897

[30] Shao M Han S Carass A Li X Blitz AM Shin J . Brain ventricle parcellation using a deep neural network: application to patients with ventriculomegaly. Neuroimage Clin. (2019) 23:101871. doi: 10.1016/j.nicl.2019.101871, 31174103 PMC6551563

[31] Kerestes R Han S Balachander S Hernandez-Castillo C Prince JL Diedrichsen J . A standardized pipeline for examining human cerebellar grey matter morphometry using structural magnetic resonance imaging. J Vis Exp. (2022) 180:340. doi: 10.3791/63340 35188124

[32] Azad R Aghdam EK Rauland A Jia Y Avval AH Bozorgpour A . Medical image segmentation review: the success of U-net. IEEE Trans Pattern Anal Mach Intell. (2024) 46:10076–95. doi: 10.1109/TPAMI.2024.3435571, 39167505

[33] Shao M. Han S. Carass A. Li X. Blitz A.M. Prince J.L. ., Shortcomings of ventricle segmentation using deep convolutional networks, Underst Interpret Mach Learn Med Image Comput Appl 11038 (2018) (2018) 79–86. doi: 10.1007/978-3-030-02628-8_9 PMC7577585 33094293

[34] Makropoulos A Counsell SJ Rueckert D. A review on automatic fetal and neonatal brain MRI segmentation. Neuroimage. (2018) 170:231–48. doi: 10.1016/j.neuroimage.2017.06.074, 28666878

[35] Drai M Testud B Brun G Hak J-F Scavarda D Girard N . Borrowing strength from adults: transferability of AI algorithms for paediatric brain and tumour segmentation. Eur J Radiol. (2022) 151:110291. doi: 10.1016/j.ejrad.2022.110291, 35405580

[36] Sharma SR Alshathri S Singh B Kaur M Mostafa RR El-Shafai W. Hybrid multilevel thresholding image segmentation approach for brain MRI. Diagnostics. (2023) 13:925. doi: 10.3390/diagnostics13050925, 36900074 PMC10000536

[37] Kotte S Pullakura RK Injeti SK. Optimal multilevel thresholding selection for brain MRI image segmentation based on adaptive wind driven optimization. Measurement. (2018) 130:340–61. doi: 10.1016/j.measurement.2018.08.007

[38] Khorram B Yazdi M. A new optimized thresholding method using ant Colony algorithm for MR brain image segmentation. J Digit Imaging. (2019) 32:162–74. doi: 10.1007/s10278-018-0111-x, 30091112 PMC6382633

[39] Tarkhaneh O Shen H. An adaptive differential evolution algorithm to optimal multi-level thresholding for MRI brain image segmentation. Expert Syst Appl. (2019) 138:112820. doi: 10.1016/j.eswa.2019.07.037

[40] Otsu N. A threshold selection method from gray-level histograms. IEEE Trans Syst Man Cybern. (1979) 9:62–6. doi: 10.1109/tsmc.1979.4310076

[41] Meng X-L Van Dyk D. The EM algorithm—an old folk-song sung to a fast new tune. J R Stat Soc Series B Stat Methodol. (1997) 59:511–67. doi: 10.1111/1467-9868.00082

[42] Greenspan H Ruf A Goldberger J. Constrained Gaussian mixture model framework for automatic segmentation of MR brain images. IEEE Trans Med Imaging. (2006) 25:1233–45. doi: 10.1109/TMI.2006.880668, 16967808

[43] Xia Y Ji Z Zhang Y. Brain MRI image segmentation based on learning local variational Gaussian mixture models. Neurocomputing. (2016) 204:189–97. doi: 10.1016/j.neucom.2015.08.125

[44] Nguyen D.M.H. Vu H.T. Ung H.Q. Nguyen B.T., 3D-brain segmentation using deep neural network and Gaussian mixture model, in: 2017 IEEE Winter Conference on Applications of Computer Vision (WACV), IEEE, (2017).

[45] Balafar MA. Gaussian mixture model based segmentation methods for brain MRI images. Artif Intell Rev. (2014) 41:429–39. doi: 10.1007/s10462-012-9317-3

[46] Schnack HG He HP Baaré WF Viergever MA Kahn RS. Automatic segmentation of the ventricular system from MR images of the human brain. NeuroImage. (2001) 14:95–104. doi: 10.1006/nimg.2001.0800, 11525342

[47] Liu J Huang S Nowinski WL. Automatic segmentation of the human brain ventricles from MR images by knowledge-based region growing and trimming. Neuroinformatics. (2009) 7:131–46. doi: 10.1007/s12021-009-9046-1, 19449142

[48] Ghafoorian M Teuwen J Manniesing R de Leeuw F-E van Ginneken B Karssemeijer N . "Student beats the teacher: deep neural networks for lateral ventricles segmentation in brain MR". In: Angelini ED Landman BA, editors. Medical Imaging 2018: Image Processing. Houston, Texas, United States: SPIE (2018). p. 744–9.

[49] Ikotun AM Ezugwu AE Abualigah L Abuhaija B Heming J. K-means clustering algorithms: a comprehensive review, variants analysis, and advances in the era of big data. Inf Sci. (2023) 622:178–210. doi: 10.1016/j.ins.2022.11.139

[50] Kong Y Wu J Yang G Zuo Y Chen Y Shu H . Iterative spatial fuzzy clustering for 3D brain magnetic resonance image supervoxel segmentation. J Neurosci Methods. (2019) 311:17–27. doi: 10.1016/j.jneumeth.2018.10.007, 30315839

[51] Barra V Boire JY. Tissue segmentation on MR images of the brain by possibilistic clustering on a 3D wavelet representation. J Magn Reson Imaging. (2000) 11:267–78. doi: 10.1002/(SICI)1522-2586(200003)11:3<267::AID-JMRI5>3.0.CO;2-8, 10739558

[52] Suckling J Sigmundsson T Greenwood K Bullmore ET. A modified fuzzy clustering algorithm for operator independent brain tissue classification of dual echo MR images. Magn Reson Imaging. (1999) 17:1065–76. doi: 10.1016/S0730-725X(99)00055-7, 10463658

[53] Makropoulos A Gousias IS Ledig C Aljabar P Serag A Hajnal JV . Automatic whole brain MRI segmentation of the developing neonatal brain. IEEE Trans Med Imaging. (2014) 33:1818–31. doi: 10.1109/TMI.2014.2322280, 24816548

[54] Pereira S Pinto A Oliveira J Mendrik AM Correia JH Silva CA. Automatic brain tissue segmentation in MR images using random forests and conditional random fields. J Neurosci Methods. (2016) 270:111–23. doi: 10.1016/j.jneumeth.2016.06.017, 27329005

[55] Kapás Z Lefkovits L Szilágyi L. "Automatic detection and segmentation of brain tumor using random Forest approach". In: Torra V Narukawa Y Navarro-Arribas G Yañez C, editors. Modeling Decisions for Artificial Intelligence. Cham: Springer (2016). p. 301–12.

[56] Lefkovits L Lefkovits S Szilágyi L. Brain tumor segmentation with optimized random forest, brainlesion: glioma, multiple sclerosis. Stroke Traumatic Brain Injuries. (2016) 10154:88–99. doi: 10.1007/978-3-319-55524-9_9

[57] McInerney T Terzopoulos D. "Deformable models in medical image analysis". In: Proceedings of the Workshop on Mathematical Methods in Biomedical Image Analysis. San Francisco, CA, USA: IEEE (1996).

[58] Jayadevappa D Kumar S Murty DS. Medical image segmentation algorithms using deformable models: a review. IETE Tech Rev. (2011) 28:248. doi: 10.4103/0256-4602.81244

[59] Talairach J. Tournoux P., Co-planar Stereotaxic Atlas of the Human Brain: 3-Dimensional Proportional System: An Approach to Cerebral Imaging, Stuttgart, New York: Cambridge University Press. (1988).

[60] Evans A.C. Collins D.L. Mills S.R. Brown E.D. Kelly R.L. Peters T.M., 3D statistical neuroanatomical models from 305 MRI volumes, in: 1993 IEEE Conference Record Nuclear Science Symposium and Medical Imaging Conference, IEEE, (2005).

[61] Ashburner J Friston KJ. Nonlinear spatial normalization using basis functions. Hum Brain Mapp. (1999) 7:254–66. doi: 10.1002/(SICI)1097-0193(1999)7:4<254::AID-HBM4>3.0.CO;2-G, 10408769 PMC6873340

[62] Guimond A Meunier J Thirion J-P. Average brain models: a convergence study. Comput Vis Image Underst. (2000) 77:192–210. doi: 10.1006/cviu.1999.0815

[63] Lorenzen P Davis B Joshi S. Unbiased atlas formation via large deformations metric mapping. Med Image Comput Comput Assist Interv. (2005) 8:411–8. doi: 10.1007/11566489_51, 16685986

[64] Zöllei L Learned-Miller E Grimson E Wells W. "Efficient population registration of 3D data". In: Liu Y Jiang T Zhang C, editors. Computer Vision for Biomedical Image Applications. Berlin: Springer Berlin Heidelberg (2005). p. 291–301.

[65] Cabezas M Oliver A Lladó X Freixenet J Cuadra MB. A review of atlas-based segmentation for magnetic resonance brain images. Comput Methods Prog Biomed. (2011) 104:e158–77. doi: 10.1016/j.cmpb.2011.07.015, 21871688

[66] Dubost F de Bruijne M Nardin M Dalca AV Donahue KL Giese A-K . Multi-atlas image registration of clinical data with automated quality assessment using ventricle segmentation. Med Image Anal. (2020) 63:101698. doi: 10.1016/j.media.2020.101698, 32339896 PMC7275913

[67] Despotović I Goossens B Philips W. MRI segmentation of the human brain: challenges, methods, and applications. Comput Math Methods Med. (2015) 2015:450341. doi: 10.1155/2015/450341 25945121 PMC4402572

[68] Toga AW Thompson PM Mori S Amunts K Zilles K. Towards multimodal atlases of the human brain. Nat Rev Neurosci. (2006) 7:952–66. doi: 10.1038/nrn2012, 17115077 PMC3113553

[69] Fawzi A Achuthan A Belaton B. Brain image segmentation in recent years: a narrative review. Brain Sci. (2021) 11:55. doi: 10.3390/brainsci11081055, 34439674 PMC8392552

[70] Lee M Kim J Ey Kim R Kim HG Oh SW Lee MK . Split-attention U-net: a fully convolutional network for robust multi-label segmentation from brain MRI. Brain Sci. (2020) 10:974. doi: 10.3390/brainsci10120974, 33322640 PMC7764312

[71] Zheng P Zhu X Guo W. Brain tumour segmentation based on an improved U-net. BMC Med Imaging. (2022) 22:199. doi: 10.1186/s12880-022-00931-1 36401207 PMC9673428

[72] Pfefferbaum A Rohlfing T Rosenbloom MJ Chu W Colrain IM Sullivan EV. Variation in longitudinal trajectories of regional brain volumes of healthy men and women (ages 10 to 85 years) measured with atlas-based parcellation of MRI. Neuroimage. (2013) 65:176–93. doi: 10.1016/j.neuroimage.2012.10.008, 23063452 PMC3516371

[73] Todd KL Brighton T Norton ES Schick S Elkins W Pletnikova O . Ventricular and periventricular anomalies in the aging and cognitively impaired brain. Front Aging Neurosci. (2017) 9:445. doi: 10.3389/fnagi.2017.00445, 29379433 PMC5771258

[74] Shprecher D Schwalb J Kurlan R. Normal pressure hydrocephalus: diagnosis and treatment. Curr Neurol Neurosci Rep. (2008) 8:371–6. doi: 10.1007/s11910-008-0058-2, 18713572 PMC2674287

[75] Espay AJ Da Prat GA Dwivedi AK Rodriguez-Porcel F Vaughan JE Rosso M . Deconstructing normal pressure hydrocephalus: Ventriculomegaly as early sign of neurodegeneration. Ann Neurol. (2017) 82:503–13. doi: 10.1002/ana.25046, 28892572

[76] Mori E Ishikawa M Kato T Kazui H Miyake H Miyajima M . Guidelines for management of idiopathic normal pressure hydrocephalus: second edition. Neurol Med Chir (Tokyo). (2012) 52:775–809. doi: 10.2176/nmc.52.775, 23183074

[77] Bradac O. Normal Pressure Hydrocephalus: Pathophysiology, Diagnosis, Treatment and Outcome. Cham, Switzerland: Springer Nature (2023).

[78] Bendella Z Purrer V Haase R Zülow S Kindler C Borger V . Brain and ventricle volume alterations in idiopathic Normal pressure hydrocephalus determined by artificial intelligence-based MRI Volumetry. Diagnostics. (2024) 14:422. doi: 10.3390/diagnostics14131422, 39001312 PMC11241572

[79] Park HY Kim M Suh CH Lee DH Shim WH Kim SJ. Diagnostic performance and interobserver agreement of the callosal angle and Evans’ index in idiopathic normal pressure hydrocephalus: a systematic review and meta-analysis. Eur Radiol. (2021) 31:5300–11. doi: 10.1007/s00330-020-07555-5, 33409775

[80] He W Fang X Wang X Gao P Gao X Zhou X . A new index for assessing cerebral ventricular volume in idiopathic normal-pressure hydrocephalus: a comparison with Evans’ index. Neuroradiology. (2020) 62:661–7. doi: 10.1007/s00234-020-02361-8, 32008047

[81] Kang K Kwak K Yoon U Lee J-M. Lateral ventricle enlargement and cortical thinning in idiopathic Normal-pressure hydrocephalus patients. Sci Rep. (2018) 8:13306. doi: 10.1038/s41598-018-31399-1, 30190599 PMC6127145

[82] Saito A Kamagata K Ueda R Nakazawa M Andica C Irie R . Ventricular volumetry and free-water corrected diffusion tensor imaging of the anterior thalamic radiation in idiopathic normal pressure hydrocephalus. J Neuroradiol. (2020) 47:312–7. doi: 10.1016/j.neurad.2019.04.003, 31034894

[83] Cogswell PM Murphy MC Senjem ML Botha H Gunter JL Elder BD . Changes in ventricular and cortical volumes following shunt placement in patients with idiopathic Normal pressure hydrocephalus. AJNR Am J Neuroradiol. (2021) 42:2165–71. doi: 10.3174/ajnr.A7323, 34674997 PMC8805754

[84] Haug JO. Pneumoencephalographic studies in mental disease. Acta Psychiatr Scand Suppl. (1962) 38:1–104. 13960992

[85] Johnstone EC Crow TJ Frith CD Husband J Kreel L. Cerebral ventricular size and cognitive impairment in chronic schizophrenia. Lancet. (1976) 2:924–6. doi: 10.1016/S0140-6736(76)90890-4, 62160

[86] Smith RC Calderon M Ravichandran GK Largen J Vroulis G Shvartsburd A . Nuclear magnetic resonance in schizophrenia: a preliminary study. Psychiatry Res. (1984) 12:137–47. doi: 10.1016/0165-1781(84)90013-1, 6591219

[87] Haijma SV Van Haren N Cahn W Koolschijn PCMP Hulshoff Pol HE Kahn RS. Brain volumes in schizophrenia: a meta-analysis in over 18 000 subjects. Schizophr Bull. (2013) 39:1129–38. doi: 10.1093/schbul/sbs118, 23042112 PMC3756785

[88] Takahashi T Suzuki M. Brain morphologic changes in early stages of psychosis: implications for clinical application and early intervention. Psychiatry Clin Neurosci. (2018) 72:556–71. doi: 10.1111/pcn.12670, 29717522

[89] Chung Y Haut KM He G van Erp TGM McEwen S Addington J . Ventricular enlargement and progressive reduction of cortical gray matter are linked in prodromal youth who develop psychosis. Schizophr Res. (2017) 189:169–74. doi: 10.1016/j.schres.2017.02.014, 28245961 PMC5572513

[90] Styner M Lieberman JA McClure RK Weinberger DR Jones DW Gerig G. Morphometric analysis of lateral ventricles in schizophrenia and healthy controls regarding genetic and disease-specific factors. Proc Natl Acad Sci USA. (2005) 102:4872–7. doi: 10.1073/pnas.0501117102, 15772166 PMC555727

[91] Manohar L Ganesan K. Detection of schizophrenia in brain MR images based on segmented ventricle region and deep belief networks. Neural Comput Applic. (2019) 31:5195–206. doi: 10.1007/s00521-018-3360-1

[92] Nuninga JO Mandl RCW Siero J Nieuwdorp W Heringa SM Boks MP . Shape and volume changes of the superior lateral ventricle after electroconvulsive therapy measured with ultra-high field MRI. Psychiatry Res Neuroimaging. (2021) 317:111384. doi: 10.1016/j.pscychresns.2021.111384, 34537602

[93] Bolin PK Gosnell SN Brandel-Ankrapp K Srinivasan N Castellanos A Salas R. Decreased brain ventricular volume in psychiatric inpatients with serotonin reuptake inhibitor treatment. Chronic Stress. (2022) 6:24705470221111092. doi: 10.1177/24705470221111092, 35859799 PMC9290100

[94] Buchsbaum MS Yang S Hazlett E Siegel BV Jr Germans M Haznedar M . Ventricular volume and asymmetry in schizotypal personality disorder and schizophrenia assessed with magnetic resonance imaging. Schizophr Res. (1997) 27:45–53. doi: 10.1016/S0920-9964(97)00087-X, 9373894

[95] Seghier ML. Laterality index in functional MRI: methodological issues. Magn Reson Imaging. (2008) 26:594–601. doi: 10.1016/j.mri.2007.10.010, 18158224 PMC2726301

[96] Manelis A Hu H Miceli R Satz S Lau R Iyengar S . The relationship between the size and asymmetry of the lateral ventricles and cortical myelin content in individuals with mood disorders. medRxiv. (2024). doi: 10.1101/2024.04.30.24306621, 38746112 PMC11092679

[97] Qiu M Liu G Zhang H Huang Y Ying S Wang J . The insular subregions topological characteristics of patients with bipolar depressive disorder. Front Psych. (2020) 11:253. doi: 10.3389/fpsyt.2020.00253, 32351411 PMC7175992

[98] Fullard K Maller JJ Welton T Lyon M Gordon E Koslow SH . Is occipital bending a structural biomarker of risk for depression and sensitivity to treatment? J Clin Neurosci. (2019) 63:55–61. doi: 10.1016/j.jocn.2019.02.007, 30827879

[99] Jack CR Jr Shiung MM Gunter JL O’Brien PC Weigand SD Knopman DS . Comparison of different MRI brain atrophy rate measures with clinical disease progression in AD. Neurology. (2004) 62:591–600. doi: 10.1212/01.WNL.0000110315.26026.EF, 14981176 PMC2730165

[100] Chou Y-Y Leporé N Avedissian C Madsen SK Parikshak N Hua X . Alzheimer’s Disease Neuroimaging Initiative, mapping correlations between ventricular expansion and CSF amyloid and tau biomarkers in 240 subjects with Alzheimer’s disease, mild cognitive impairment and elderly controls. Neuroimage. (2009) 46:394–410. doi: 10.1016/j.neuroimage.2009.02.015, 19236926 PMC2696357

[101] Nestor SM Rupsingh R Borrie M Smith M Accomazzi V Wells JL . Alzheimer’s Disease Neuroimaging Initiative, ventricular enlargement as a possible measure of Alzheimer’s disease progression validated using the Alzheimer's disease neuroimaging initiative database. Brain. (2008) 131:2443–54. doi: 10.1093/brain/awn146, 18669512 PMC2724905

[102] Lundervold AJ Vik A Lundervold A. Lateral ventricle volume trajectories predict response inhibition in older age-a longitudinal brain imaging and machine learning approach. PLoS One. (2019) 14:e0207967. doi: 10.1371/journal.pone.0207967, 30939173 PMC6445521

[103] Nigro S Filardi M Tafuri B Nicolardi M De Blasi R Giugno A . Deep learning-based approach for brainstem and ventricular MR planimetry: application in patients with progressive supranuclear palsy. Radiol Artif Intell. (2024) 6:e230151. doi: 10.1148/ryai.230151 38506619 PMC11140505

[104] Fernandez-Lozano S Fonov V Schoemaker D Pruessner J Potvin O Duchesne S . Automatization and validation of the hippocampal-to-ventricle ratio in a clinical sample. bioRxiv. (2024). doi: 10.1101/2024.04.12.588928 PMC12314924 40747973

[105] Steed TC Treiber JM Taha B Engin HB Carter H Patel KS . Glioblastomas located in proximity to the subventricular zone (SVZ) exhibited enrichment of gene expression profiles associated with the cancer stem cell state. J Neuro-Oncol. (2020) 148:455–62. doi: 10.1007/s11060-020-03550-4, 32556864

[106] Steed TC Treiber JM Brandel MG Patel KS Dale AM Carter BS . Quantification of glioblastoma mass effect by lateral ventricle displacement. Sci Rep. (2018) 8:2827. doi: 10.1038/s41598-018-21147-w, 29434275 PMC5809591

[107] Pura JA Hamilton AM Vargish GA Butman JA Linguraru MG. "Automated segmentation of ventricles from serial brain MRI for the quantification of volumetric changes associated with communicating hydrocephalus in patients with brain tumor". In: Weaver JB Molthen RC, editors. Medical Imaging 2011: Biomedical Applications in Molecular, Structural, and Functional Imaging. Lake Buena Vista (Orlando), Florida, United States: SPIE (2011).

[108] Branson HM. Normal myelination: a practical pictorial review. Neuroimaging Clin N Am. (2013) 23:183–95. doi: 10.1016/j.nic.2012.12.001 23608684

[109] Richter L Fetit AE. Accurate segmentation of neonatal brain MRI with deep learning. Front Neuroinform. (2022) 16:1006532. doi: 10.3389/fninf.2022.1006532, 36246394 PMC9554654

[110] Devi CN Chandrasekharan A Sundararaman VK Alex ZC. Neonatal brain MRI segmentation: a review. Comput Biol Med. (2015) 64:163–78. doi: 10.1016/j.compbiomed.2015.06.016, 26189155

[111] Hashimoto H Takemoto O Nishimoto K Moriguchi G Nakamura M Chiba Y. Normal growth curve of choroid plexus in children: implications for assessing hydrocephalus due to choroid plexus hyperplasia. J Neurosurg Pediatr. (2023) 32:627–37. doi: 10.3171/2023.7.PEDS23218, 37724840

[112] Xenos C Sgouros S Natarajan K. Ventricular volume change in childhood. J Neurosurg. (2002) 97:584–90. doi: 10.3171/jns.2002.97.3.0584, 12296642

[113] Szentimrey Z de Ribaupierre S Fenster A Ukwatta E. Automated 3D U-net based segmentation of neonatal cerebral ventricles from 3D ultrasound images. Med Phys. (2022) 49:1034–46. doi: 10.1002/mp.15432, 34958147

[114] Qiu W Chen Y Kishimoto J de Ribaupierre S Chiu B Fenster A . Automatic segmentation approach to extracting neonatal cerebral ventricles from 3D ultrasound images. Med Image Anal. (2017) 35:181–91. doi: 10.1016/j.media.2016.06.038, 27428629

[115] Davies MW Swaminathan M Chuang SL Betheras FR. Reference ranges for the linear dimensions of the intracranial ventricles in preterm neonates. Arch Dis Child Fetal Neonatal Ed. (2000) 82:F218–23. doi: 10.1136/fn.82.3.F218, 10794790 PMC1721078

[116] Ballabh P. Intraventricular hemorrhage in premature infants: mechanism of disease. Pediatr Res. (2010) 67:1–8. doi: 10.1203/pdr.0b013e3181c1b176, 19816235 PMC2799187

[117] Murphy BP Inder TE Rooks V Taylor GA Anderson NJ Mogridge N . Posthaemorrhagic ventricular dilatation in the premature infant: natural history and predictors of outcome. Arch Dis Child Fetal Neonatal Ed. (2002) 87:F37–41. doi: 10.1136/fn.87.1.F37, 12091289 PMC1721419

[118] Gholipour A Akhondi-Asl A Estroff JA Warfield SK. Multi-atlas multi-shape segmentation of fetal brain MRI for volumetric and morphometric analysis of ventriculomegaly. Neuroimage. (2012) 60:1819–31. doi: 10.1016/j.neuroimage.2012.01.128, 22500924 PMC3329183

[119] Qiu W Yuan J Rajchl M Kishimoto J Chen Y de Ribaupierre S . 3D MR ventricle segmentation in pre-term infants with post-hemorrhagic ventricle dilatation (PHVD) using multi-phase geodesic level-sets. Neuroimage. (2015) 118:13–25. doi: 10.1016/j.neuroimage.2015.05.099, 26070262

[120] Quon JL Han M Kim LH Koran ME Chen LC Lee EH . Artificial intelligence for automatic cerebral ventricle segmentation and volume calculation: a clinical tool for the evaluation of pediatric hydrocephalus. J Neurosurg Pediatr. (2021) 27:131–8. doi: 10.3171/2020.6.PEDS20251, 33260138 PMC9707365

[121] Tabrizi P.R. Obeid R. Mansoor A. Ensel S. Cerrolaza J.J. Penn A. ., Cranial ultrasound-based prediction of post hemorrhagic hydrocephalus outcome in premature neonates with intraventricular hemorrhage, Annual International Conference of the IEEE Engineering in Medicine and Biology Society (2017) 169–172. 10.1109/EMBC.2017.8036789 29059837

[122] Huang KT McNulty J Hussein H Klinger N Chua MMJ Ng PR . Automated ventricular segmentation and shunt failure detection using convolutional neural networks. Sci Rep. (2024) 14:22166. doi: 10.1038/s41598-024-73167-4, 39333724 PMC11436930

[123] Isıklar S Turan Ozdemir S Ozkaya G Ozpar R Parlak M. Morphological evaluation of the normal and hydrocephalic third ventricle on cranial magnetic resonance imaging in children: a retrospective study. Pediatr Radiol. (2023) 53:282–96. doi: 10.1007/s00247-022-05475-8, 35994062

[124] American Psychiatric Association. Diagnostic and Statistical Manual of Mental Disorders (DSM-5). Washington, District of Columbia, USA: American Psychiatric Publishing (2021).

[125] Shiohama T Ortug A Warren JLA Valli B Levman J Faja SK . Small nucleus Accumbens and large cerebral ventricles in infants and toddlers prior to receiving diagnoses of autism Spectrum disorder. Cereb Cortex. (2022) 32:1200–11. doi: 10.1093/cercor/bhab283, 34455432 PMC8924432

[126] Solomon O Palnitkar T Patriat R Braun H Aman J Park MC . Deep-learning based fully automatic segmentation of the globus pallidus interna and externa using ultra-high 7 tesla MRI. Hum Brain Mapp. (2021) 42:2862–79. doi: 10.1002/hbm.25409, 33738898 PMC8127160

[127] Patriat R Cooper SE Duchin Y Niederer J Lenglet C Aman J . Individualized tractography-based parcellation of the globus pallidus pars interna using 7T MRI in movement disorder patients prior to DBS surgery. Neuroimage. (2018) 178:198–209. doi: 10.1016/j.neuroimage.2018.05.048 29787868 PMC6046264

[128] Kim J Duchin Y Shamir RR Patriat R Vitek J Harel N . Automatic localization of the subthalamic nucleus on patient-specific clinical MRI by incorporating 7 T MRI and machine learning: application in deep brain stimulation. Hum Brain Mapp. (2019) 40:679–98. doi: 10.1002/hbm.24404, 30379376 PMC6519731

[129] Svanera M Benini S Bontempi D Muckli L. CEREBRUM-7T: fast and fully volumetric brain segmentation of 7 tesla MR volumes. Hum Brain Mapp. (2021) 42:5563–80. doi: 10.1002/hbm.25636, 34598307 PMC8559470

[130] Kulkarni AV Guha A Lozano A Bernstein M. Incidence of silent hemorrhage and delayed deterioration after stereotactic brain biopsy. J Neurosurg. (1998) 89:31–5. doi: 10.3171/jns.1998.89.1.0031, 9647169

[131] Lau BL Vijian K Liew DNS Wong ASH. Factors affecting diagnostic yield in stereotactic biopsy for brain lesions: a 5-year single-center series. Neurosurg Rev. (2022) 45:1473–80. doi: 10.1007/s10143-021-01671-6, 34628562

[132] Seaman SC Li L Menezes AH Dlouhy BJ. Fourth ventricle roof angle as a measure of fourth ventricle bowing and a radiographic predictor of brainstem dysfunction in Chiari malformation type I. J Neurosurg Pediatr. (2021) 28:260–7. doi: 10.3171/2021.1.PEDS20756, 34171843

[133] Robusto J Coulthard LG Yates C Mantha S Campbell R. Fourth ventricular roof angle does not predict surgical outcome in paediatric patients with Chiari I malformation. Childs Nerv Syst. (2024) 40:4083–7. doi: 10.1007/s00381-024-06614-2, 39349774

[134] Xiao Y Liu Y Wang Z He K Zhang Z Chen S . Combined cerebrospinal fluid hydrodynamics and fourth ventricle outlet morphology to improve predictive efficiency of prognosis for Chiari malformation type I decompression. World Neurosurg. (2023) 176:e208–18. doi: 10.1016/j.wneu.2023.05.031, 37187345

[135] Taha BR. Evaluating linear heuristics for ventricular volume in healthy adults using a fully automated algorithm: implications for defining the Normal. Neurosurgery. (2024) 96:693–9. doi: 10.1227/neu.0000000000003132, 39115316

[136] Sjaastad O Nordvik A. The corpus callosal angle in the diagnosis of cerebral ventricular enlargement. Acta Neurol Scand. (1973) 49:396–406. doi: 10.1111/j.1600-0404.1973.tb01312.x, 4542888

[137] Kulkarni AV Drake JM Kestle JRW Mallucci CL Sgouros S Constantini S. Canadian Pediatric neurosurgery study group, predicting who will benefit from endoscopic third ventriculostomy compared with shunt insertion in childhood hydrocephalus using the ETV success score. J Neurosurg Pediatr. (2010) 6:310–5. doi: 10.3171/2010.8.PEDS103, 20887100

[138] Verhey LH Kulkarni AV Reeder RW Riva-Cambrin J Jensen H Pollack IF . Hydrocephalus clinical research network, a re-evaluation of the endoscopic third Ventriculostomy success score: a hydrocephalus clinical research network study. J Neurosurg Pediatr. (2024) 33:417–27. doi: 10.3171/2023.12.PEDS23401, 38335514

[139] Dlouhy BJ Capuano AW Madhavan K Torner JC Greenlee JDW. Preoperative third ventricular bowing as a predictor of endoscopic third ventriculostomy success. J Neurosurg Pediatr. (2012) 9:182–90. doi: 10.3171/2011.11.PEDS11495 22295925

[140] Foroughi M Wong A Steinbok P Singhal A Sargent MA Cochrane DD. Third ventricular shape: a predictor of endoscopic third ventriculostomy success in pediatric patients. J Neurosurg Pediatr. (2011) 7:389–96. doi: 10.3171/2011.1.PEDS10461, 21456911

[141] O’Hayon BB Drake JM Ossip MG Tuli S Clarke M. Frontal and occipital horn ratio: a linear estimate of ventricular size for multiple imaging modalities in pediatric hydrocephalus. Pediatr Neurosurg. (1998) 29:245–9. doi: 10.1159/000028730, 9917541

[142] Ragan DK Cerqua J Nash T McKinstry RC Shimony JS Jones BV . The accuracy of linear indices of ventricular volume in pediatric hydrocephalus: technical note. J Neurosurg Pediatr. (2015) 15:547–51. doi: 10.3171/2014.10.PEDS14209, 25745953 PMC4558898

[143] Karnan M. Selvanayaki K. Improved implementation of brain MR image segmentation using meta heuristic algorithms, in: 2010 IEEE International Conference on Computational Intelligence and Computing Research, IEEE (2010).

[144] Kurdi SZ Ali MH Jaber MM Saba T Rehman A Damaševičius R. Brain tumor classification using meta-heuristic optimized convolutional neural networks. J Pers Med. (2023) 13:181. doi: 10.3390/jpm13020181, 36836415 PMC9965936

[145] Kaur D Singh S Mansoor W Kumar Y Verma S Dash S . Computational intelligence and metaheuristic techniques for brain tumor detection through IoMT-enabled MRI devices. Wirel Commun Mob Comput. (2022) 2022:1–20. doi: 10.1155/2022/1519198

