# Supplementary material for: A review of applications of automated ventricular parcellation from magnetic resonance imaging of the brain
Source: Front Neurol. 2026 Apr 1;16:1639381. doi: 10.3389/fneur.2025.1639381 (PMC13080440; doi:10.3389/fneur.2025.1639381)
Supplement: Supplementary file 1 [file Data_Sheet_1.pdf]

Model name	Operating system	Segmentation method	URL
vol2Brain	Browser-based	Multi-atlas, patch	https://volbrain.net/services/vol2Brain
MALPEM	Linux-based systems	Multi-atlas	https://github.com/ledigchr/MALPEM
ALVIN	Windows, MacOS, Linux-based systems	Binary mask	https://www.nitrc.org/projects/alvin_lv/
FreeSurfer	Linux-based systems, MacOS	Atlas	https://github.com/freesurfer/freesurfer
FastSurfer	Linux-based systems	Autoencoder neural network architecture (U-net)	https://github.com/Deep-MI/FastSurfer
SLANT	Linux-based systems	Mixed patch-based with fully convolutional neural network	https://github.com/MASILab/SLANTbrainSeg
QuickNAT2	Windows, MacOS, Linux-based systems	Autoencoder neural network architecture (U-net)	https://github.com/ai-med/QuickNATv2
BrainSegNet	Linux-based systems	Mixed patch-based with fully convolutional neural network	https://github.com/Parth-nXp/BrainSegNet
MindGlide	Linux-based systems	Autoencoder neural network	https://github.com/MS-PINPOINT/mindGlide

		architecture (U-net)	
OpenMAP-T1	Linux-based systems	Autoencoder neural network architecture (U-net)	https://github.com/OishiLab/OpenMAP-T1
SynthSeg	Linux-based systems	Autoencoder neural network architecture (U-net)	https://github.com/BBillot/SynthSeg
VoxHRNet	Linux-based systems	Modified fully convolutional neural network	https://github.com/microsoft/VoxHRNet
